# Emphysematous Cystitis and Septic Shock: A Multidisciplinary Approach to Managing a Life-Threatening Urological Infection

**DOI:** 10.7759/cureus.73685

**Published:** 2024-11-14

**Authors:** Teresa Frazão, Ana Patrícia Brito, Joana Cerdeira Frazão, Jorge Cotter

**Affiliations:** 1 Internal Medicine, Unidade Local de Saúde do Alto Ave, Guimarães, PRT; 2 Internal Medicine, Hospital Senhora da Oliveira, Guimarães, PRT

**Keywords:** bladder, diabetes mellitus, emphysematous cystitis, infection, klebsiella pneumoniae, septic shock, urinary tract infection

## Abstract

Emphysematous cystitis (EC) is a rare, life-threatening urinary tract infection (UTI) characterized by gas formation within the bladder wall and lumen. It predominantly occurs in patients with poorly controlled diabetes. We present a case of a 61-year-old male with poorly controlled diabetes and chronic alcoholism who was admitted in a comatose state due to severe septic shock secondary to EC. Initial management included mechanical ventilation, vasopressor support, and broad-spectrum antibiotics. Cultures revealed *Klebsiella pneumoniae* resistant to the initial therapy, prompting a switch to Meropenem. With prompt multidisciplinary intervention, the patient gradually recovered, was successfully extubated, and was transferred from the ICU by day 5. He was discharged from the hospital on day 17 with full clinical recovery. This case highlights the critical role of early diagnosis, appropriate antimicrobial therapy, and intensive supportive care in managing severe cases of EC with multiorgan failure.

## Introduction

Emphysematous cystitis (EC) is a rare but serious urinary tract infection (UTI) characterized by the presence of gas within the bladder wall and lumen, accounting for less than 1% of all UTIs [1]. It is most frequently caused by *Escherichia coli* and *Klebsiella pneumoniae*, which produce gas through fermentation and its accumulation due to factors like tissue ischemia, local necrosis, and host immunosuppression [1-3]. The condition was first described by Bailey in 1961 and is predominantly seen in patients with risk factors such as poorly controlled diabetes mellitus, chronic UTIs, long-term catheter use, neurogenic bladder, or immunosuppression [4,5]. Approximately 50-70% of cases are associated with diabetes, which promotes gas formation through mechanisms like glycosuria and impaired neutrophil function [6]. Although uncommon, EC can progress rapidly and lead to life-threatening complications if not diagnosed and managed promptly [7]. Symptoms include dysuria, hematuria, suprapubic pain, fever, and, in more severe cases, can even lead to sepsis [1]. Due to nonspecific symptoms, diagnosis is often delayed, particularly in high-risk individuals [7]. The gold standard for diagnosis is computed tomography (CT) imaging, which clearly shows gas within the bladder wall and any complications [5]. Management typically involves broad-spectrum antibiotics, glycemic control, and bladder drainage [3]. Surgical intervention is rarely necessary but may be required in severe cases such as bladder rupture or necrosis [6]. Prognosis largely depends on the promptness of diagnosis and treatment. Mortality rates range from 7% to 14%, with a higher risk of complications in severe cases [2]. Potential complications include bladder rupture, emphysematous pyelonephritis, and sepsis [1].

## Case presentation

The patient is a 61-year-old male who was brought to the emergency room in a comatose state with a Glasgow Coma Scale (GCS) of 3. The family reported a history of abdominal pain localized to the right hypochondrium. The patient presented with a sudden decrease in consciousness, prompting emergency medical services to transfer him to the hospital. He had complained of diffuse abdominal pain with predominant discomfort in the right hypochondrium over the past few days. There was no recent history of fever, nausea, vomiting, or change in bowel habits. The patient also showed signs of respiratory distress with bradypnea on arrival. On abdominal examination, there was mild distension with tenderness localized to the right hypochondrium, without rebound tenderness or guarding.

Prior to this presentation, the patient had the following conditions: arterial hypertension, managed with antihypertensive therapy but with inconsistent follow-up; poorly controlled type 2 diabetes, with elevated HbA1c levels in recent tests and known diabetic complications, including diabetic nephropathy with chronic kidney disease stage 3b according to the Kidney Disease: Improving Global Outcomes (KDIGO) classification, and diabetic retinopathy. Apart from that, the patient had a long-standing history of alcohol use disorder, contributing to poor self-care and difficulty managing his comorbidities.

**Figure 1:**
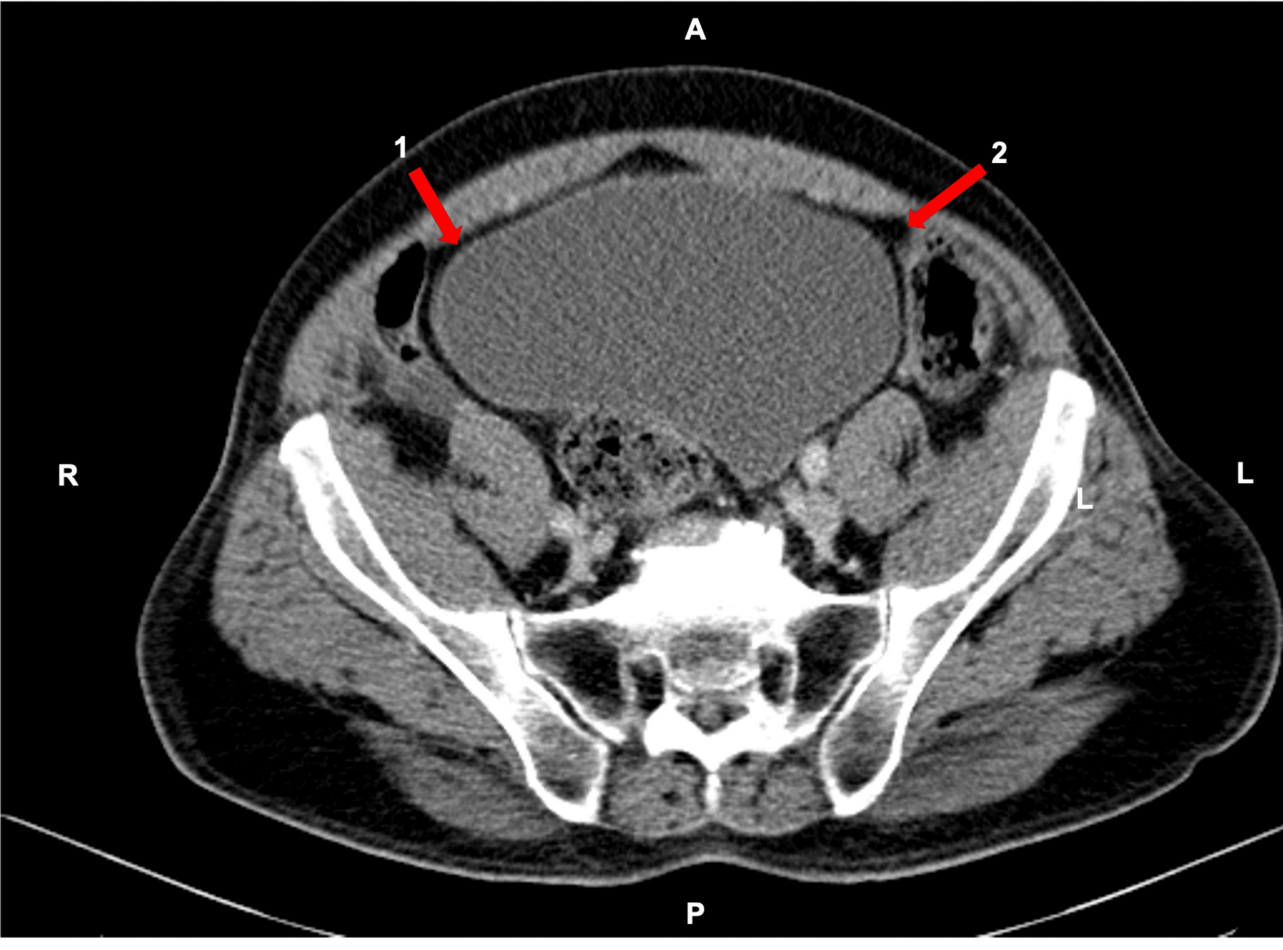
Axial section on contrast-enhanced CT of the abdomen and pelvis Bladder wall intramural gas favoring the diagnosis of EC. (1) and (2): Gas bubbles in the bladder wall. (A) Anterior. (B) Posterior. EC, emphysematous cystitis; CT, computed tomography

Lab tests revealed hyperlacticaemia (8.0 mmol/L), severe thrombocytopenia (47,000/µL), acute kidney injury grade 3 (serum creatinine: 3.1 mg/dL), euvolemic hyponatremia of 122 mEq/L, elevation of gamma-glutamyl transferase (543 U/L) and alkaline phosphatase (329 U/L), and C- reactive protein (CRP) of 117 mg/L (elevated). Urinalysis showed an elevated leukocyte count and a positive nitrite test. Imaging studies of CT (Figure 1) revealed a distended bladder with intramural and intraluminal gas, consistent with EC, and no evidence of bowel perforation or free intra-abdominal air.

Head CT had no relevant findings. Immediate endotracheal intubation was performed due to the patient's low GCS and respiratory failure. The patient received intravenous fluids and vasopressors to stabilize blood pressure. The patient was diagnosed with septic shock secondary to EC. The infection rapidly progressed, leading to a critical clinical state with multiorgan failure involving the neurologic, hematologic, liver, and renal systems.

Given the patient’s critical condition, he was promptly admitted to the Intensive Care Unit (ICU) for aggressive hemodynamic and respiratory support, including mechanical ventilation, continuous vasopressor support, broad-spectrum antibiotics (Piperacillin-Tazobactam 4.5 g IV every 6 hours), and continuous monitoring.

On day 2, the admission urine and blood cultures grew *Klebsiella pneumoniae*, which was found to be resistant to the empiric therapy of Piperacillin-Tazobactam given. Consequently, antibiotic therapy was switched to Meropenem according to the sensitivity testing. The patient’s condition gradually stabilized under the new regimen. By day 3, his hemodynamics were stable, and vasopressor support was discontinued.

On day 4, he was successfully extubated without complications, showing improved neurological status. The patient was transferred from the ICU to an intermediate care unit due to his clinical improvement and decreasing CRP levels, indicating a favorable response to the targeted antibiotic treatment. By day 10 (the eighth day of Meropenem therapy), the patient’s blood and urine cultures were negative for bacterial growth, confirming effective infection control. He was then transferred to the Urology ward for further monitoring and management. The patient continued to show steady improvement, and by day 17, he was discharged from the hospital with full resolution of the EC and multiorgan failure.

Given the severity of the initial presentation and his comorbid conditions, the patient was advised to have a close outpatient follow-up with the Urology department. Long-term management of his poorly controlled diabetes and regular monitoring for recurrent UTIs were emphasized to reduce the risk of recurrence and complications.

## Discussion

EC is a potentially life-threatening urological emergency and complication of UTIs and it is most commonly seen in patients with poorly controlled diabetes [4]. In this case, the patient’s history of poorly managed type 2 diabetes placed him at high risk for this aggressive infection. Additionally, the presence of diabetic nephropathy and retinopathy further indicated a high degree of end-organ damage and long-standing metabolic derangement, making this patient particularly vulnerable to severe infections.

Early recognition and diagnosis of EC are crucial because of its potential for rapid progression to severe sepsis, septic shock, and multiorgan failure, as seen in this patient. The initial presentation in a comatose state with a GCS of 3 and evidence of respiratory failure underscored the severity of the patient’s systemic response to infection. Multiorgan failure, including acute kidney injury (AKI), thrombocytopenia, and liver dysfunction, suggested that the patient was already in a state of advanced sepsis upon arrival. Imaging studies play a pivotal role in diagnosing EC, as the hallmark finding is the presence of intramural and intraluminal gas in the bladder on abdominal CT.

The initial empirical therapy with broad-spectrum antibiotics was appropriate given the patient’s critical condition. This case highlights the importance of prompt culture acquisition and susceptibility testing in guiding appropriate antimicrobial therapy, especially in the context of multidrug-resistant organisms.

The multidisciplinary approach involving critical care and internal medicine specialists and urologists was essential in stabilizing the patient. This collaborative management allowed for rapid intervention and optimization of supportive therapies, ultimately leading to a favorable outcome.

## Conclusions

This case demonstrates a rare and life-threatening presentation of emphysematous cystitis complicated by septic shock and multiorgan failure. It highlights the importance of early recognition, timely imaging, and targeted antibiotic therapy in managing this severe urological emergency. The patient’s favorable outcome despite a critical initial presentation was achieved through a combination of aggressive supportive care and tailored antimicrobial therapy, showcasing the potential for recovery in even the most severe cases of EC when managed appropriately.
